# X-ray crystal and computational structural study of (*E*)-2-[(2-chloro­phenyl)­iminometh­yl]-4-methoxy­phenol

**DOI:** 10.1107/S1600536808021958

**Published:** 2008-07-23

**Authors:** Arzu Özek, Orhan Büyükgüngör, Çiğdem Albayrak, Mustafa Odabaşoğlu

**Affiliations:** a Department of Physics, Ondokuz Mayıs University, TR-55139 Samsun, Turkey; b Department of Chemistry, Ondokuz Mayıs University, TR-55139 Samsun, Turkey

## Abstract

In the mol­ecule of the title compound, C_14_H_12_ClNO, the two aromatic rings are oriented at a dihedral angle of 12.28 (7)°. An intra­molecular O—H⋯N hydrogen bond results in the formation of a nearly planar six-membered ring, which is oriented with respect to the aromatic rings at dihedral angles of 0.18 (5) and 12.10 (6)°. In the crystal structure, weak inter­molecular C—H⋯O hydrogen bonds link the mol­ecules into chains along the *c* axis. There is a C—H⋯π contact between the methyl group and the chloro­phenyl ring and a π–π contact between the two benzene rings [centroid–centroid distance = 3.866 (1) Å].

## Related literature

For related literature, see: Özek *et al.* (2007 ▶); Odabaşoğlu, Büyükgüngör *et al.* (2007 ▶); Odabaşoğlu, Arslan *et al.* (2007 ▶); Albayrak *et al.* (2005 ▶); Elerman *et al.* (1995 ▶); Frisch *et al.* (2004 ▶). For general background, see: Friesner (2005 ▶); Liu *et al.* (2004 ▶).
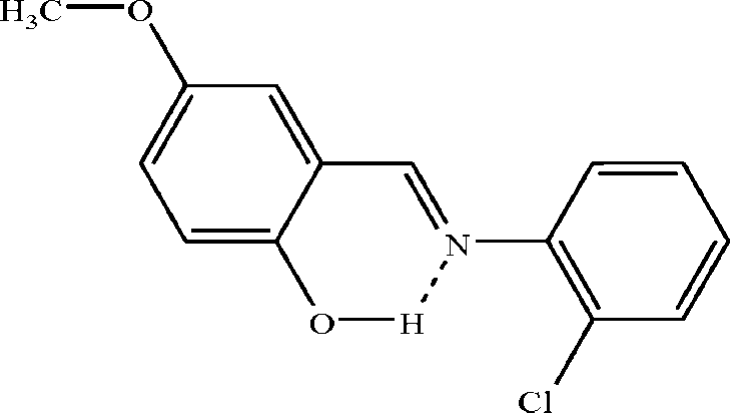


## Experimental

### 

#### Crystal data

C_14_H_12_ClNO_2_
*M* *_r_* = 261.70
Monoclinic, 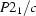

*a* = 13.2348 (9) Å
*b* = 8.4701 (4) Å
*c* = 12.0115 (8) Å
β = 112.846 (5)°
*V* = 1240.86 (13) Å^3^
*Z* = 4
Mo *K*α radiation
μ = 0.30 mm^−1^
*T* = 296 K
0.56 × 0.40 × 0.11 mm

#### Data collection

Stoe IPDSII diffractometer
Absorption correction: integration (*X-RED32*; Stoe & Cie, 2002 ▶) *T*_min_ = 0.851, *T*_max_ = 0.966
15517 measured reflections
2441 independent reflections
1977 reflections with *I* > 2σ(*I*)
*R*_int_ = 0.042

#### Refinement

*R*[*F* ^2^ > 2σ(*F* ^2^)] = 0.031
*wR*(*F* ^2^) = 0.089
*S* = 1.05
2441 reflections
167 parameters
H atoms treated by a mixture of independent and constrained refinement
Δρ_max_ = 0.13 e Å^−3^
Δρ_min_ = −0.21 e Å^−3^

### 

Data collection: *X-AREA* (Stoe & Cie, 2002 ▶); cell refinement: *X-AREA*; data reduction: *X-RED32* (Stoe & Cie, 2002 ▶); program(s) used to solve structure: *SHELXS97* (Sheldrick, 2008 ▶); program(s) used to refine structure: *SHELXL97* (Sheldrick, 2008 ▶); molecular graphics: *ORTEP-3 for Windows* (Farrugia, 1997 ▶); software used to prepare material for publication: *WinGX* (Farrugia, 1999 ▶).

## Supplementary Material

Crystal structure: contains datablocks I, global. DOI: 10.1107/S1600536808021958/hk2494sup1.cif

Structure factors: contains datablocks I. DOI: 10.1107/S1600536808021958/hk2494Isup2.hkl

Additional supplementary materials:  crystallographic information; 3D view; checkCIF report

## Figures and Tables

**Table 1:** Hydrogen-bond geometry (Å, °) *Cg*2 is the centroid of the C9–C14 ring.

*D*—H⋯*A*	*D*—H	H⋯*A*	*D*⋯*A*	*D*—H⋯*A*
O1—H1⋯N1	0.80 (2)	1.85 (2)	2.5896 (16)	152 (2)
C8—H8⋯O1^i^	0.93	2.58	3.4960 (19)	169
C7—H7b⋯*Cg*2^ii^	0.96	2.90	3.682	139

Symmetry codes: (i) 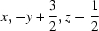; (ii) 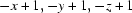.

**Table 2:** Selected geometric parameters (Å, °) calculated with X-ray, AM1, PM3, HF and DFT

Parameters	X-ray	AM1	PM3	HF^*a*^	DFT/B3LYP^*a*^
C8—N1	1.278 (17)	1.292	1.302	1.262	1.294
C2—O1	1.357 (17)	1.366	1.355	1.332	1.341
C1—C6	1.407 (18)	1.412	1.406	1.408	1.416
C1—C8	1.447 (19)	1.465	1.478	1.463	1.446
C1—C2	1.399 (19)	1.404	1.408	1.392	1.418
N1—C9	1.408 (17)	1.408	1.427	1.402	1.399
C9—C10	1.392 (18)	1.417	1.402	1.393	1.409
C10—Cl1	1.734 (14)	1.699	1.680	1.741	1.755
C5—O2	1.369 (17)	1.385	1.385	1.354	1.369
					
C9—C10—Cl1	120.02 (10)	120.869	120.554	120.163	119.783
C6—C5—O2	125.3 (15)	124.874	125.684	125.547	125.410
C6—C1—C8	119.24 (13)	116.155	117.987	117.891	119.224
C9—N1—C8	122.41 (12)	121.909	122.720	120.140	121.089
C14—C9—N1	124.73 (12)	123.114	123.424	122.078	122.787
N1—C8—C1	120.75 (13)	123.585	119.187	123.458	122.291
N1—C9—C10	117.64 (12)	118.844	116.913	119.874	119.562
					
C8—C1—C2—O1	0.6 (2)	−0.034	0.012	−0.194	−0.175
C6—C5—O2—C7	−1.7 (2)	0.543	−0.485	0.568	0.096
C10—C9—N1—C8	−165.93 (12)	−147.255	−179.982	−134.578	−144.790
N1—C8—C1—C6	176.91 (12)	176.946	−179.997	179.409	179.781
C1—C8—N1—C9	−178.32 (11)	−179.082	179.999	−178.064	−176.682

Note: (*a*) 6-31G(d,p).

**Fig. 1.**
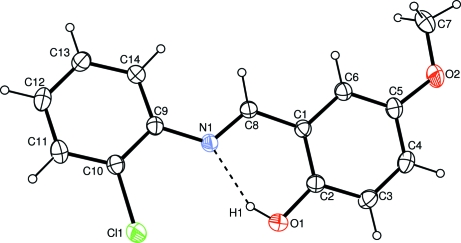
The molecular structure of the title molecule, with the atom-numbering scheme. Displacement ellipsoids are drawn at the 20% probability level. Hydrogen bond is shown as dashed line.

**Fig. 2.**
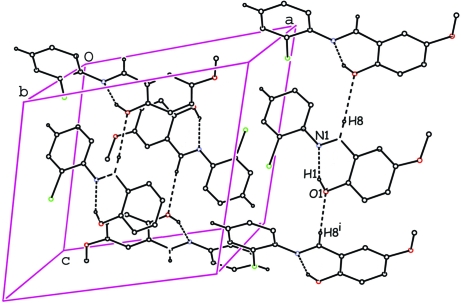
A partial packing diagram of (I), showing the formation of C(5) chains [symmetry code: (i) x, -y + 3/2, z + 1/2]. Hydrogen bonds are shown as dashed lines. H atoms not involved in hydrogen bonding have been omitted for clarity.

**Fig. 3.**
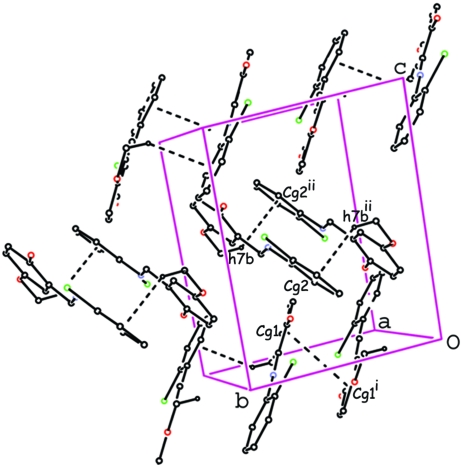
A partial packing diagram of (I), showing the formation of the C—H···π and π···π interactions [symmetry codes; (i) 1 - x, 1 - y, - z; (ii) 1 - x, 1 - y, 1 - z]. Hydrogen bonds are shown as dashed lines. H atoms not involved in hydrogen bonding have been omitted for clarity.

## supplementary crystallographic information

### Comment

The present work is part of a structural study of Schiff bases Özek *et al.*,  2007; Odabaşoğlu, Büyükgüngör *et al.*, 2007; Odabaşoğlu, Arslan *et al.*, 2007). We report herein the crystal structure of the title compound, (I).

In general, O-hydroxy Schiff bases exhibit two possible tautomeric forms, the phenol-imine (or benzenoid) and keto-amine (or quinoid) forms. Depending on the tautomers, two types of intra-molecular hydrogen bonds are possible: O-H···N in benzenoid and N-H···O in quinoid tautomers. The H atom in (I) is located on atom O1, thus the phenol-imine tautomer is favored over the keto -amine form, as indicated by the C2-O1, C8-N1, C1-C8 and C1-C2 bonds (Fig. 1, Table 2). The O1-C2 bond has single-bond character, whereas the N1-C8 bond has a high degree of double-bond character as in 2-(3-methoxysalicylideneamino)-1H -benzimidazolemonohydrate, (II) [where the corresponding values are C-O = 1.357 (2) Å and C-N = 1.285 (2) Å] (Albayrak *et al.*,  2005).

It is known that Schiff bases may exhibit thermochromism or photochromism, depending on the planarity or non-planarity of the molecule, respectively. Therefore, one can expect thermochromic properties in (I) caused by the planarity of the molecule; the dihedral angle between rings A (C1-C6) and B (C9-C14) is 12.28 (7)°. The intramolecular O-H···N hydrogen bond (Table 1) results in the formation of a nearly planar six-membered ring C (O1/H1/N1/C1/C2/C8), in which it is oriented with respect to rings A and B at dihedral angles of A/C = 0.18 (5)° and B/C = 12.10 (6)°. So, it is coplanar with the adjacent ring A. It generates an S(6) ring motif. The O1···N1 [2.589 (2) Å] distance is comparable to those observed for analogous ones in N-(2-hydroxyphenyl)salicylaldimine, (III) [2.675 (7) Å; Elerman *et al.*, 1995] and in three(E)-2-[(bromophenyl)iminomethyl]-4-methoxyphenols, (IV) [2.603 (2), 2.638 (7) and 2.577 (4) Å;Özek *et al.*,  2007].

In the crystal structure, weak intermolecular C-H···O hydrogen bonds (Table 1) results in the formation of C(5) chains along the c axis (Fig. 2), in which they may be effective in the stabilization of the structure. A C—H···π contact (Table 1) between the methyl group and ring B and a π—π contact (Fig. 3) between the symmetry related A rings Cg1···Cg1^i^ [symmetry code: (i) 1 - x, 1 - y, - z, where Cg1 is the centroid of ring A] further stabilize the structure, with centroid-centroid distance of 3.866 (1) Å.

Ab-*initio* Hartree-Fock (HF), density-functional theory (DFT) and semi-empirical (AM1 and PM3) calculations and full-geometry optimizations were performed by means of *GAUSSIAN* 03 W package (Frisch *et al.*, 2004). The selected bond lengths and angles together with the torsion angles are compared with the obtained ones from semi-empirical, ab-initio HF and DFT/B3LYP methods (Table 2). We observe an acceptable general agreement between them. Although the DFT molecular orbital theory was considered as the most accurate method for geometry optimization for free and complex ligands (Friesner, 2005; Liu *et al.*,  2004), the HF method led to better results in regard to the bond lengths and angles.

### Experimental

The title compound was prepared by refluxing a mixture of a solution containing 5-methoxysalicylaldehyde (0.5 g 3.3 mmol) in ethanol (20 ml) and a solution containing 2-chloroaniline (0.420 g 3.3 mmol) in ethanol (20 ml). The reaction mixture was stirred for 1 h, under reflux. The crystals suitable for X-ray analysis were obtained from ethanol by slow evaporation (yield; 75%; m.p. 393-394 K).

### Refinement

H1 atom (for OH) was located in difference syntheses and refined isotropically [O-H = 0.80 (2) Å and U_iso_(H) = 0.082 (6) Å^2^]. The remaining H atoms were positioned geometrically, with C-H = 0.93 and 0.96 Å for aromatic and methyl H, respectively, and constrained to ride on their parent atoms with U_iso_(H) = 1.2U_eq_(C).

### Crystal data

**Table:**

C_14_H_12_ClNO_2_	*F*_000_ = 544
*M**_r_* = 261.70	*D*_x_ = 1.401 Mg m^−^^3^
Monoclinic, *P*2_1_/*c*	Mo *K*α radiation λ = 0.71073 Å
Hall symbol: -P 2ybc	Cell parameters from 15517 reflections
*a* = 13.2348 (9) Å	θ = 2.0–28.0º
*b* = 8.4701 (4) Å	µ = 0.30 mm^−^^1^
*c* = 12.0115 (8) Å	*T* = 296 K
β = 112.846 (5)º	Prismatic plate, red
*V* = 1240.86 (13) Å^3^	0.56 × 0.40 × 0.11 mm
*Z* = 4	

### Data collection

**Table:**

Stoe IPDSII diffractometer	2441 independent reflections
Radiation source: fine-focus sealed tube	1977 reflections with *I* > 2σ(*I*)
Monochromator: plane graphite	*R*_int_ = 0.042
Detector resolution: 6.67 pixels mm^-1^	θ_max_ = 26.0º
*T* = 296 K	θ_min_ = 2.9º
ω scans	*h* = −16→16
Absorption correction: integration(X-RED32; Stoe & Cie, 2002)	*k* = −10→10
*T*_min_ = 0.851, *T*_max_ = 0.966	*l* = −14→14
15517 measured reflections	

### Refinement

**Table:**

Refinement on *F*^2^	Secondary atom site location: difference Fourier map
Least-squares matrix: full	Hydrogen site location: inferred from neighbouring sites
*R*[*F*^2^ > 2σ(*F*^2^)] = 0.031	H atoms treated by a mixture of independent and constrained refinement
*wR*(*F*^2^) = 0.089	*w* = 1/[σ^2^(*F*_o_^2^) + (0.0523*P*)^2^ + 0.0594*P*] where *P* = (*F*_o_^2^ + 2*F*_c_^2^)/3
*S* = 1.05	(Δ/σ)_max_ = 0.001
2441 reflections	Δρ_max_ = 0.13 e Å^−^^3^
167 parameters	Δρ_min_ = −0.21 e Å^−^^3^
Primary atom site location: structure-invariant direct methods	Extinction correction: none

### Special details

**Table:**

Experimental. 336 frames, detector distance = 80 mm
Geometry. All e.s.d.'s (except the e.s.d. in the dihedral angle between two l.s. planes) are estimated using the full covariance matrix. The cell e.s.d.'s are taken into account individually in the estimation of e.s.d.'s in distances, angles and torsion angles; correlations between e.s.d.'s in cell parameters are only used when they are defined by crystal symmetry. An approximate (isotropic) treatment of cell e.s.d.'s is used for estimating e.s.d.'s involving l.s. planes.
Refinement. Refinement of F^2^ against ALL reflections. The weighted R-factor wR and goodness of fit S are based on F^2^, conventional R-factors R are based on F, with F set to zero for negative F^2^. The threshold expression of F^2^ > 2sigma(F^2^) is used only for calculating R-factors(gt) etc. and is not relevant to the choice of reflections for refinement. R-factors based on F^2^ are statistically about twice as large as those based on F, and R- factors based on ALL data will be even larger.

### Fractional atomic coordinates and isotropic or equivalent isotropic displacement parameters (Å^2^)

**Table:**

	*x*	*y*	*z*	*U*_iso_*/*U*_eq_	
Cl1	0.11627 (3)	0.76700 (5)	0.55086 (4)	0.06604 (15)	
O1	0.40106 (9)	0.86945 (14)	0.69518 (11)	0.0623 (3)	
H1	0.3554 (17)	0.822 (2)	0.641 (2)	0.082 (6)*	
O2	0.77703 (8)	0.90497 (15)	0.60329 (11)	0.0728 (3)	
N1	0.31117 (9)	0.70603 (13)	0.49903 (10)	0.0454 (3)	
C1	0.49336 (10)	0.80152 (15)	0.56335 (12)	0.0445 (3)	
C2	0.49138 (11)	0.87192 (16)	0.66786 (13)	0.0494 (3)	
C3	0.58388 (13)	0.95011 (19)	0.74523 (14)	0.0599 (4)	
H3	0.5831	0.9978	0.8146	0.072*	
C4	0.67628 (12)	0.95805 (19)	0.72077 (14)	0.0619 (4)	
H4	0.7377	1.0109	0.7739	0.074*	
C5	0.67990 (11)	0.88837 (17)	0.61772 (14)	0.0547 (3)	
C6	0.58916 (11)	0.81123 (16)	0.53942 (13)	0.0501 (3)	
H6	0.5909	0.7650	0.4700	0.060*	
C7	0.78611 (14)	0.8339 (2)	0.50131 (18)	0.0726 (5)	
H7A	0.7313	0.8768	0.4291	0.087*	
H7B	0.7756	0.7220	0.5037	0.087*	
H7C	0.8576	0.8544	0.5018	0.087*	
C8	0.39782 (11)	0.72290 (15)	0.47745 (12)	0.0457 (3)	
H8	0.3995	0.6842	0.4057	0.055*	
C9	0.21762 (10)	0.62666 (15)	0.41915 (12)	0.0443 (3)	
C10	0.12031 (11)	0.64486 (15)	0.43678 (13)	0.0479 (3)	
C11	0.02522 (11)	0.56821 (18)	0.36454 (15)	0.0595 (4)	
H11	−0.0391	0.5828	0.3773	0.071*	
C12	0.02614 (12)	0.47049 (19)	0.27390 (15)	0.0637 (4)	
H12	−0.0376	0.4188	0.2249	0.076*	
C13	0.12142 (13)	0.4492 (2)	0.25577 (14)	0.0629 (4)	
H13	0.1220	0.3819	0.1948	0.075*	
C14	0.21635 (12)	0.52647 (17)	0.32687 (13)	0.0548 (3)	
H14	0.2801	0.5114	0.3130	0.066*	

### Atomic displacement parameters (Å^2^)

**Table:**

	*U*^11^	*U*^22^	*U*^33^	*U*^12^	*U*^13^	*U*^23^
C1	0.0411 (7)	0.0489 (7)	0.0414 (7)	−0.0018 (5)	0.0136 (5)	0.0020 (5)
C2	0.0508 (7)	0.0523 (7)	0.0453 (7)	−0.0019 (6)	0.0190 (6)	0.0008 (6)
C3	0.0626 (9)	0.0646 (9)	0.0469 (8)	−0.0067 (7)	0.0153 (7)	−0.0092 (7)
C4	0.0500 (8)	0.0653 (9)	0.0580 (9)	−0.0116 (7)	0.0075 (7)	−0.0067 (7)
C5	0.0406 (7)	0.0569 (8)	0.0624 (9)	−0.0023 (6)	0.0154 (6)	0.0041 (7)
C6	0.0452 (7)	0.0553 (7)	0.0495 (8)	−0.0023 (6)	0.0180 (6)	−0.0003 (6)
C7	0.0531 (9)	0.0848 (11)	0.0877 (12)	−0.0008 (8)	0.0358 (9)	0.0109 (10)
C8	0.0453 (7)	0.0523 (7)	0.0395 (7)	−0.0034 (5)	0.0164 (5)	0.0004 (5)
C9	0.0422 (7)	0.0460 (6)	0.0422 (7)	−0.0028 (5)	0.0135 (5)	0.0062 (5)
C10	0.0451 (7)	0.0468 (7)	0.0511 (8)	−0.0005 (5)	0.0179 (6)	0.0037 (6)
C11	0.0410 (7)	0.0591 (8)	0.0747 (10)	−0.0029 (6)	0.0184 (7)	0.0021 (7)
C12	0.0500 (8)	0.0664 (9)	0.0622 (10)	−0.0138 (7)	0.0082 (7)	−0.0034 (7)
C13	0.0663 (10)	0.0682 (9)	0.0526 (9)	−0.0154 (7)	0.0214 (7)	−0.0101 (7)
C14	0.0533 (8)	0.0621 (8)	0.0525 (8)	−0.0088 (6)	0.0246 (6)	−0.0040 (7)
N1	0.0406 (6)	0.0517 (6)	0.0430 (6)	−0.0029 (4)	0.0153 (5)	0.0017 (5)
O1	0.0597 (7)	0.0781 (7)	0.0560 (6)	−0.0107 (5)	0.0301 (5)	−0.0142 (6)
O2	0.0437 (6)	0.0865 (8)	0.0880 (9)	−0.0134 (5)	0.0255 (6)	−0.0087 (7)
Cl1	0.0595 (2)	0.0677 (2)	0.0810 (3)	−0.00680 (17)	0.0382 (2)	−0.01591 (19)

### Geometric parameters (Å, °)

**Table:**

O1—H1	0.80 (2)	C7—H7C	0.9600
C1—C2	1.3992 (19)	C8—N1	1.2781 (17)
C1—C6	1.4071 (18)	C8—H8	0.9300
C1—C8	1.4470 (19)	C9—C14	1.3908 (19)
C2—O1	1.3572 (17)	C9—C10	1.3925 (18)
C2—C3	1.385 (2)	C9—N1	1.4082 (17)
C3—C4	1.366 (2)	C10—C11	1.3822 (19)
C3—H3	0.9300	C10—Cl1	1.7342 (14)
C4—C5	1.389 (2)	C11—C12	1.371 (2)
C4—H4	0.9300	C11—H11	0.9300
C5—O2	1.3694 (17)	C12—C13	1.372 (2)
C5—C6	1.370 (2)	C12—H12	0.9300
C6—H6	0.9300	C13—C14	1.379 (2)
C7—O2	1.411 (2)	C13—H13	0.9300
C7—H7A	0.9600	C14—H14	0.9300
C7—H7B	0.9600		
			
C2—O1—H1	105.4 (15)	O2—C7—H7C	109.5
C5—O2—C7	117.81 (12)	H7A—C7—H7C	109.5
C8—N1—C9	122.41 (12)	H7B—C7—H7C	109.5
C2—C1—C6	119.38 (12)	N1—C8—C1	120.75 (13)
C2—C1—C8	121.36 (12)	N1—C8—H8	119.6
C6—C1—C8	119.24 (13)	C1—C8—H8	119.6
O1—C2—C3	118.51 (13)	C14—C9—C10	117.58 (12)
O1—C2—C1	122.37 (12)	C14—C9—N1	124.73 (12)
C3—C2—C1	119.10 (13)	C10—C9—N1	117.64 (12)
C4—C3—C2	120.75 (14)	C11—C10—C9	121.54 (13)
C4—C3—H3	119.6	C11—C10—Cl1	118.44 (11)
C2—C3—H3	119.6	C9—C10—Cl1	120.02 (10)
C3—C4—C5	120.96 (13)	C12—C11—C10	119.69 (14)
C3—C4—H4	119.5	C12—C11—H11	120.2
C5—C4—H4	119.5	C10—C11—H11	120.2
O2—C5—C6	125.30 (15)	C11—C12—C13	119.81 (13)
O2—C5—C4	115.35 (13)	C11—C12—H12	120.1
C6—C5—C4	119.35 (13)	C13—C12—H12	120.1
C5—C6—C1	120.45 (14)	C12—C13—C14	120.77 (15)
C5—C6—H6	119.8	C12—C13—H13	119.6
C1—C6—H6	119.8	C14—C13—H13	119.6
O2—C7—H7A	109.5	C13—C14—C9	120.61 (14)
O2—C7—H7B	109.5	C13—C14—H14	119.7
H7A—C7—H7B	109.5	C9—C14—H14	119.7
			
C6—C1—C2—O1	178.75 (13)	C4—C5—C6—C1	−0.4 (2)
C8—C1—C2—O1	0.6 (2)	C1—C8—N1—C9	−178.32 (11)
C6—C1—C2—C3	0.2 (2)	C14—C9—N1—C8	16.7 (2)
C8—C1—C2—C3	−177.93 (13)	C10—C9—N1—C8	−165.93 (12)
C2—C1—C6—C5	0.2 (2)	C14—C9—C10—C11	−0.9 (2)
C8—C1—C6—C5	178.38 (13)	N1—C9—C10—C11	−178.38 (12)
C2—C1—C8—N1	−4.9 (2)	C14—C9—C10—Cl1	179.58 (10)
C6—C1—C8—N1	176.91 (12)	N1—C9—C10—Cl1	2.05 (16)
O1—C2—C3—C4	−178.98 (14)	C10—C9—C14—C13	0.3 (2)
C1—C2—C3—C4	−0.4 (2)	N1—C9—C14—C13	177.61 (13)
C2—C3—C4—C5	0.1 (2)	C9—C10—C11—C12	0.6 (2)
C3—C4—C5—O2	179.83 (15)	Cl1—C10—C11—C12	−179.78 (12)
C3—C4—C5—C6	0.3 (2)	C10—C11—C12—C13	0.2 (2)
C6—C5—O2—C7	−1.7 (2)	C11—C12—C13—C14	−0.7 (2)
C4—C5—O2—C7	178.80 (14)	C12—C13—C14—C9	0.5 (2)
O2—C5—C6—C1	−179.95 (13)		

### Hydrogen-bond geometry (Å, °)

**Table:**

*D*—H···*A*	*D*—H	H···*A*	*D*···*A*	*D*—H···*A*
O1—H1···N1	0.80 (2)	1.85 (2)	2.5896 (16)	152 (2)
C8—H8···O1^i^	0.93	2.58	3.4960 (19)	169
C7—H7b···Cg2^ii^	0.96	2.90	3.682	139.00

Symmetry codes: (i) *x*, −*y*+3/2, *z*−1/2; (ii) −*x*+1, −*y*+1, −*z*+1.

### Table 2 Selected geometric parameters (Å, °) calculated with X-ray, AM1, PM3, HF and DFT for (I)

**Table:**

Parameters	X-ray	AM1	PM3	HF^a^	DFT/B3LYP^a^
C8—N1	1.278 (17)	1.292	1.302	1.262	1.294
C2—O1	1357 (17)	1.366	1.355	1.332	1.341
C1—C6	1.407 (18)	1.412	1.406	1.408	1.416
C1—C8	1.447 (19)	1.465	1.478	1.463	1.446
C1—C2	1.399 (19)	1.404	1.408	1.392	1.418
N1—C9	1.408 (17)	1.408	1.427	1.402	1.399
C9—C10	1.392 (18)	1.417	1.402	1.393	1.409
C10—Cl1	1.734 (14)	1.699	1.680	1.741	1.755
C5—-O2	1.369 (17)	1.385	1.385	1.354	1.369
					
C9—C10—Cl1	120.02 (10)	120.869	120.554	120.163	119.783
C6—C5—O2	125.3 (15)	124.874	125.684	125.547	125.410
C6—C1—C8	119.24 (13)	116.155	117.987	117.891	119.224
C9—N1—C8	122.41 (12)	121.909	122.720	120.140	121.089
C14—C9—N1	124.73 (12)	123.114	123.424	122.078	122.787
N1—C8—C1	120.75 (13)	123.585	119.187	123.458	122.291
N1—C9—C10	117.64 (12)	118.844	116.913	119.874	119.562
					
C8—C1—C2—O1	0.6 (2)	-0.034	0.012	-0.194	-0.175
C6—C5—O2—C7	-1.7 (2)	0.543	-0.485	0.568	0.096
C10—C9—N1—C8	-165.93 (12)	-147.255	-179.982	-134.578	-144.790
N1—C8—C1—C6	176.91 (12)	176.946	-179.997	179.409	179.781
C1—C8—N1—C9	-178.32 (11)	-179.082	179.999	-178.064	-176.682

Notes: (a) 6-31G(d,p).
